# The presence of cerebellar B cell aggregates is associated with a specific chemokine profile in the cerebrospinal fluid in a mouse model of multiple sclerosis

**DOI:** 10.1186/s12974-023-02695-z

**Published:** 2023-01-30

**Authors:** Verena Schropp, Rittika Chunder, Barbara Dietel, Sabine Tacke, Stefanie Kuerten

**Affiliations:** 1 Medical Faculty, Institute of Neuroanatomy, University of Bonn, 53115 Bonn, Germany; 2 Institute of Anatomy and Cell Biology, Friedrich-Alexander-Universität Erlangen-Nürnberg (FAU), 91054 Erlangen, Germany; 3 Department of Cardiology and Angiology, Friedrich-Alexander-Universität Erlangen-Nürnberg (FAU), Erlangen University Hospital, 91054 Erlangen, Germany

**Keywords:** B cell aggregates, Chemokines, CSF, EAE, MP4

## Abstract

**Background:**

The presence of meningeal ectopic lymphoid structures (ELS) in a subgroup of patients diagnosed with secondary progressive multiple sclerosis (SPMS) corresponds to a pronounced cortical inflammation and an aggravated disease course. In MP4-induced experimental autoimmune encephalomyelitis (EAE), a mouse model of multiple sclerosis (MS), B cell aggregates develop in the central nervous system (CNS) in the chronic stage of the disease. Therefore, the model is suitable for studying key molecules of ELS development and maintenance. Here, we investigated whether there is a specific cytokine and chemokine signature in paired cerebrospinal fluid (CSF) and serum samples associated with the presence of cerebellar B cell and T cell pathology and B cell aggregates of MP4-immunized mice.

**Methods:**

Paired CSF and serum samples were collected from the *cisterna magna* and periphery of MP4-immunized mice at the chronic stage of disease. A control group with mice immunized only with the adjuvant (vehicle) was included in the study. A selected panel of 34 cytokines and chemokines were measured by MAGPIX® for both cohorts. For the assessment of B cell and T cell infiltration, immunohistochemical staining was performed and analyzed using light microscopy. To detect specific chemokine receptors additional staining was conducted.

**Results:**

While we detected several upregulated cytokines and chemokines in the CSF of MP4-immunized mice independent of the extent of B cell and T cell pathology compared to vehicle-immunized mice, C-C motif chemokine ligand (CCL)-1 was associated with high B cell and T cell infiltration. Furthermore, the level of certain chemokines, including CCL1, CCL5, CCL7, CCL12, CCL22 and C-X-C motif chemokine ligand (CXCL)-13, was significantly increased (*p* < 0.05) in MP4-immunized mice showing a high number of B cell aggregates. While C-C motif chemokine receptor (CCR)5 had a ubiquitous expression independent of the extent of B cell and T cell pathology, C-X-C motif chemokine receptor (CXCR)-5 and CXCR6 expression was specifically associated with high B cell and T cell pathology.

**Conclusion:**

Our data suggest that multiple cytokines and chemokines are involved in the pathophysiology of MP4-induced EAE. Furthermore, the presence of B cell aggregates was associated with a specific chemokine profile in the CSF, which might be useful for predicting the presence of these aggregates without the necessity to histologically screen the CNS tissue.

**Supplementary Information:**

The online version contains supplementary material available at 10.1186/s12974-023-02695-z.

## Background

Multiple sclerosis (MS) is a neuroinflammatory autoimmune disease of the central nervous system (CNS). Historically, the pathogenesis of MS has been considered to be driven by T cells [1]. However, with the development and success of CD20^+^ cell-depleting therapies, B cells have been catapulted into taking the center stage in the field of MS research [2]. The involvement of B cells in the pathophysiology of MS also comes from neuropathological studies of autopsied brain tissue from MS patients [3, 4]. For instance, meningeal ectopic lymphoid structures (ELS) have been detected in up to 40% of patients with secondary progressive MS (SPMS). These ELS displayed characteristics similar to that of secondary lymphoid organs (SLO), including the presence of follicular dendritic cells (FDC) and proliferating B cells. The association of these structures with a high degree of cortical pathology and a more severe disease course indicates a potential clinical relevance of ELS in MS progression [5].

However, the development and maintenance of ELS in MS is poorly understood. One study has shown that B-cell activating factor of the tumor necrosis factor (TNF) family (BAFF), which is important for long-term survival and persistence of B cells, can be locally produced by astrocytes in MS lesions thereby possibly aiding in the survival of B cells in the inflamed CNS [6]. Meanwhile, CXCL13 was found to be elevated in the CSF of relapsing–remitting MS patients (RRMS) and correlated with the presence of B cells and plasmablasts [7]. This link between the elevated CXCL13 level and the increased number of B cells in the CSF also suggests that CXCL13 may be an important B cell chemoattractant in the inflamed MS brain. Additionally, CXCL13 has been detected in ELS of SPMS patients [5, 8]. Finally, SPMS patients with high meningeal inflammation and grey matter pathology showed higher levels of TNF, lymphotoxin (LT)-α and CXCL13 in the CSF compared to patients with less pathology [9].

Owing to the limited access to well-characterized CNS tissue containing ELS from MS patients and corresponding CSF samples, several studies have focussed on EAE, the animal model of MS, to identify key molecules and mechanisms involved in ELS formation and maintenance [10]. For example, T_H_17 cells have been suggested to initiate the development of ELS in the CNS [11]. There is also evidence that a combination of T_H_17 cell-related cytokines, like interleukin (IL)-17 and IL-22, as well as LT signaling are involved in the ELS formation process [12]. Other studies using animal models have identified molecules like interferon (IFN)-γ, TNF and CXCL13 to be involved in the development and maintenance of these structures [13–15].

Here we focused on MP4-induced EAE, a mouse model of MS, which is characterized by the development of B cell aggregates especially in the chronic stage of the disease [16, 17]. Using this model, we set out to identify relevant cytokines and chemokines, which may play a key role in the formation and maintenance of B cell aggregates, by analyzing paired CSF and serum samples in the chronic stage of MP4-induced EAE. To this end, we modified a method to collect CSF samples from mice [18], performed cytokine/chemokine multiplex assays and systematically characterized the extent of B cell and T cell pathology in the cerebellum, where pathology predominantly develops in the MP4 model [17]. Our data reveal a specific chemokine profile associated with the presence of B cell aggregates in the CNS of MP4-immunized mice.

## Materials and methods

### Mice

Female 7-week-old wild-type (WT) C67BL/6 (B6) mice were purchased from Charles River Laboratories (Wilmington, MA, USA). Animals were kept under specific pathogen-free conditions at the Preclinical Experimental Center for Animals (PETZ) of the Franz-Penzoldt Center (FPZ) of the University Hospital Erlangen with unrestricted access to water and a standard rodent diet (ssniff Spezialdiäten, Soest, Germany). Special care such as ClearH_2_O HydroGel (ClearH_2_O, Portland, ME, USA) was provided for mice displaying paralytic symptoms. All animal experiments were approved by the Regierung von Unterfranken (approval number 55.2-2531.01-91/14) and performed in accordance with the German law on the protection of animals, the “Principles of laboratory animal care” (NIH publication no. 86–23, revised 1985) and the ARRIVE (Animal Research: Reporting of In Vivo Experiments) guidelines.

### EAE induction and assessment of the disease course

All animals were between 10 to 15 weeks of age at the time of immunization. For EAE induction, incomplete Freund’s adjuvant (IFA) was prepared by mixing mineral oil (Merck, Darmstadt, Germany) and mannide monooleate (Merck) at a ratio of 9:1. For the preparation of complete Freund’s adjuvant (CFA), *Mycobacterium tuberculosis* H37 Ra (BD Biosciences, San Jose, CA, USA) was added to IFA at a concentration of 5 mg/ml. MP4 (Alexion Pharmaceuticals, Boston, MA, USA) was emulsified in CFA and each mouse received a total dose of 200 μg of MP4 in a volume of 200 μl which was subcutaneously injected into both sides of the flank. Vehicle mice (control group) received a total volume of 200 μl CFA and phosphate-buffered saline (PBS) (Merck) at a ratio of 1:1. Both the MP4-immunized cohort and the vehicle mice additionally received 100 ng of pertussis toxin (Hooke Laboratories Inc., Lawrence, MA, USA) by intraperitoneal injection at the day of immunization and 24 h later. To study the level of chemokine/cytokine changes in the CSF and periphery associated with the chronic stage of the disease (chronic MP4-induced EAE), mice were scored daily over a period of approximately 65 days after immunization and killed at 66.13 ± 1.83 days. Vehicle mice were killed at day 62 following immunization. For assessment of the disease course, we used the standard EAE scoring system: no symptoms (0), floppy tail (1), partial hind limb weakness (2), full hind limb paralysis (3), quadriplegia (4) and moribund (5). Increments of 0.25 were used to account for symptoms that were not clearly defined by the five categories. However, mice with a disease score ≥ 3 for more than 5 days were killed and excluded from the study.

### Preparation of sample material

#### Serum

Up to seven drops of blood were collected from the tail vein of each mouse one day prior to killing. Blood was allowed to clot at room temperature (RT) for 30–45 min and serum was collected by centrifugation at 1000 × *g* for 15 min at 4 °C. After carefully transferring the serum, another centrifugation step at 10,000 × *g* for 10 min at 4 °C was performed. Serum samples were immediately aliquoted and stored at − 80 °C until further analysis.

#### CSF

**Fig. 1:**
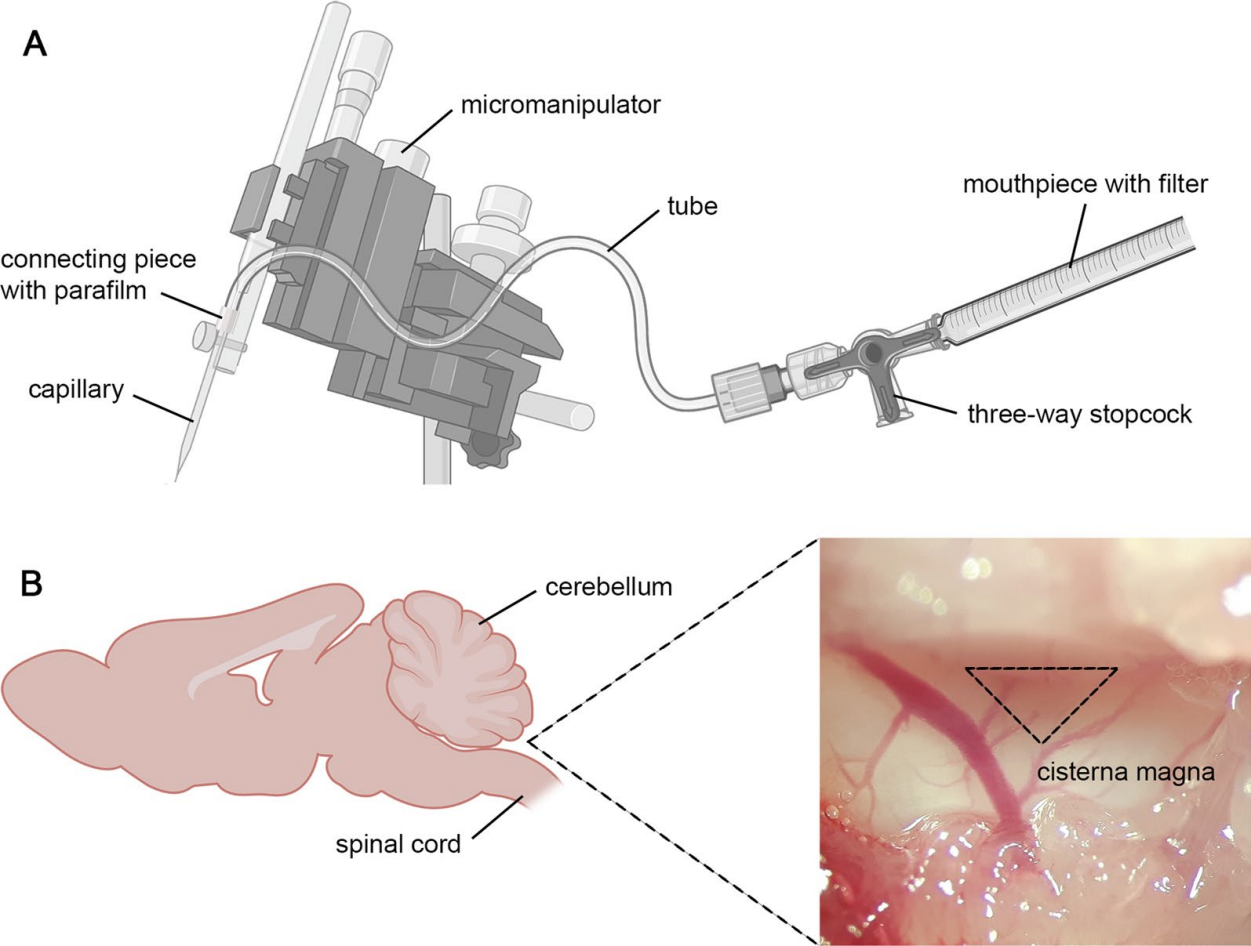
Procedure for the collection of cerebrospinal fluid from the *cisterna magna*. **A** The device for collecting CSF consisted of different components as displayed. **B** The CSF-filled *cisterna magna* was located at the back of the head and appeared as a dark triangle under the microscope. *CSF* cerebrospinal fluid

CSF sampling was adapted from the method of Boris Šakić using a self-constructed device (Fig. 1A) [18]. Briefly, a 1-ml syringe was shortened, and a ball of cotton was inserted as a filter. The syringe with the filter, which served as the mouthpiece, was fixed on a micromanipulator (Narishige Scientific Instrument Lab., Tokyo, Japan) using a three-way stopcock (Fig. 1A). Mice were killed with carbon dioxide (CO_2_) and placed in a prone position on the micromanipulator device. A sagittal incision of the skin at the occiput was made and the cranial bone was carefully removed. With the help of a microscope, the subcutaneous tissue, neck muscles and ligaments were separated to allow access to the *cisterna magna*, which is an extended cavern of the subarachnoid space filled with CSF (Fig. 1B). The *dura mater* of the *cisterna magna* was carefully pierced using a long glass capillary with an outer diameter of 1 mm and a narrowed tip. This allowed the CSF to flow into the capillary. Care was taken to adjust the capillary using the micromanipulator to precisely puncture the *cisterna magna* and avoid blood contamination. To obtain the maximum volume of CSF (range between 2.3 µl and 16 µl), this procedure was repeated until the *cisterna magna* was empty. After collection, each sample was briefly centrifuged, the supernatant was aliquoted and immediately stored at − 80 °C.

#### Cerebellum

Following CSF withdrawal, the whole brain was removed, and the cerebellum was carefully dissected using a scalpel. Tissue fixation of each cerebellum was done with 4% paraformaldehyde (PFA) solution for 48 h at 4 °C on a shaker. Following fixation, the tissues were washed in phosphate (PO_4_) buffer, dehydrated and subsequently embedded in paraffin. Serial sections were cut at a thickness of 5 µm and every fifth section (30 sections per mouse) was stained for B cells and T cells to assess pathology. Created with BioRender.com. 

### Immunohistochemistry and assessment of pathology

#### B cell and T cell staining

B cell and T cell staining of the cerebellar tissue sections was done according to the protocol previously described by our group with slight modifications [19]. Briefly, sections were deparaffinized using xylene and rehydrated in a descending series of isopropanol solution. Antigen retrieval was performed by boiling the sections in 0.01 M sodium citrate buffer (pH 6.0) for 15 min. Tissue sections were washed with Tris-buffered saline (TBS)-T (TBS + 0.1% Tween 20) between every incubation and antibodies were diluted according to Table 1. Sections were blocked for 30 min at RT followed by an incubation with an anti-CD3 antibody for 1 h at RT. Subsequently, a biotinylated anti-rabbit antibody was added, and the sections were incubated for 1 h at RT. Streptavidin coupled to alkaline phosphatase (AP) (Vector Laboratories, Burlingame, CA, USA) diluted in TBS (1:500) was applied on the sections for 30 min at RT. The sections were developed for 40 min at RT using the Vector BCIP/NBT Kit (Vector Laboratories) until blue stained CD3^+^ cells were visible under a light microscope. An anti-CD45R (B220) antibody was used to detect B cells on the same sections. Following an overnight incubation at 4 °C with the second primary antibody, a biotinylated anti-rat antibody was applied on the tissue sections for 1 h at RT. Subsequently, the sections were incubated with streptavidin-horseradish peroxide (HRP) (1:2000; Abcam, Cambridge, UK) for 35 min at RT and developed for 15 min at RT by the DAB Substrate Kit (Vector Laboratories) to obtain a brown staining product. The sections were finally counterstained with Nuclear Fast Red before dehydration and coverslipped using Entellan® Neu (Merck).

**Table 1:** Antibodies used for immunohistochemistry. Anti-mouse CD3, Rabbit/IgG, clone SP162, dilution 1:150, company Abcam, Cambridge, UK

Antibody	Species/isotype	Conjugate	Clone	Dilution	Company
Anti-mouse CCR5	Rabbit/ IgG		Polyclonal	1:125	Novus Biologicals, Littleton, CO, USA
Anti-mouse CD3	Rat/ IgG1		CD3-12	1:100	Abcam, Cambridge, UK
Anti-mouse CD45R (B220)	Rat / IgG2a, κ		RA3-6B2	1:250	Thermo Fisher Scientific, Waltham, MA, USA
Anti-mouse CXCR5	Rabbit		Polyclonal	1:500	Merck, Darmstadt, Deutschland
Anti-mouse CXCR6	Rabbit/ IgG		Polyclonal	1:150	Novus Biologicals, Littleton, CO, USA
Anti-mouse F4/80	Rat/ IgG2b		CI:A3-1	1:100	Abcam, Cambridge, UK
Anti-rabbit IgG (H + L)	Goat, IgG	Biotin	Polyclonal	1:500	Abcam, Cambridge, UK
Anti-rat IgG (H + L)	Goat	Biotin	Polyclonal	1:500	Abcam, Cambridge, UK
Anti-rabbit IgG (H + L)	Donkey	Cyanine (Cy)™3	Polyclonal	1:300	Jackson Immuno Research Labs, West Grove, PA, USA

#### B cell and T cell quantification

An Eclipse E200 light microscope (Nikon, Tokyo, Japan) was used to assess B cell and T cell pathology in the cerebellar sections of the mice. The following categories of pathology were defined: B cell infiltrates (B cells: ≥ 10), B cell aggregates (B cells: ≥ 20; clustered), T cell infiltrates (T cells: ≥ 10; B cells: < 10) and T cell clusters (T cells: ≥ 20; clustered).

#### Chemokine receptor staining

To stain for chemokine receptors, tissue sections were deparaffinized and antigen retrieval was done as mentioned above. Slides were washed between every incubation step with TBS-T and sections were incubated in a humidified chamber. Subsequently, slides were blocked with 1% bovine serum albumin (BSA) in TBS-T for 1 h at RT. All primary and secondary antibodies were diluted in 0.1% BSA (Table 1). Incubation with anti-CCR5, anti-CXCR5 or anti-CXCR6 antibody was performed overnight at 4 °C. For CCR5 staining, the secondary antibody incubation and development were carried out as mentioned above for the CD3^+^ T cell staining with a slight modification. The sections were developed only for 10 min using the Vector BCIP/NBT Kit (Vector Laboratories). For CXCR5 and CXCR6 staining, the tissue was incubated with a Cy™3-conjugated anti-rabbit secondary antibody (Jackson Immuno Research Labs, West Grove, PA, USA) for 2 h at RT in the dark and a sequential double-staining was performed by incubating the sections with either an anti-B220, anti-CD3 or anti-F4/80 antibody overnight at 4 °C. Slides were incubated with a biotinylated anti-rat antibody for 2 h at RT followed by another incubation step with streptavidin conjugated to Cy™2 (1:200; Jackson Immuno Research Labs) for 45 min at RT in the dark. All sections were counterstained using the Fluoroshield mounting medium with 4',6-diamidino-2-phenylindole (DAPI) (Abcam). A technical negative control was included for each staining where the slide was incubated with secondary antibody only (Additional file 1).

Immunofluorescent images were taken and scanning of the slides was done using a Leica DMi8 inverted microscope equipped with the Thunder Imaging software (Leica Camera Ag, Wetzlar, Germany).

### Luminex chemokine and cytokine assay

A MAGPIX® Luminex (Luminex Corporation, Austin, TX, USA) platform was used for chemokine and cytokine quantification in the serum and CSF in collaboration with the research group of Molecular and Experimental Cardiology at the Translational Research Center (TRC), University Hospital Erlangen.

To analyze the chemokines and cytokines in paired CSF and serum samples, a combination of the Bio-Plex Pro Mouse Chemokine Panel 31-Plex Kit and the Bio-Plex Pro Mouse Cytokines IL-21, IL-22 and IL-23 (Bio-Rad Laboratories Inc., Hercules, CA, USA) was used and the experiments were conducted following the manufacturer's instructions. A Bio-Plex Pro Mouse T_H_17 cytokine standard (Bio-Rad Laboratories Inc.) was included in addition to the cytokine standards provided in the Bio-Plex Pro Mouse Chemokine Panel 31-Plex Kit. Prior to MAGPIX® measurement, each sample was centrifuged and diluted in dilution buffer (provided in the kit). Every serum sample was diluted 1:5 and the dilution range for CSF was between 1:14 and 1:22, dependent on the amount obtained from each mouse. While serum was measured in duplicates, this was not the case with every CSF samples due to the restricted volume. Wells with > 1 analyte that was not measurable were excluded from the analysis. Additionally, a co-efficient of variation (CV) value of above 20 and a bead count under 50 were considered as exclusion criteria.

### Statistical analysis

Statistical analysis was performed using GraphPad Prism (9.2.0). Gaussian distribution of every dataset was checked using the Shapiro–Wilk normality test. Ordinary one-way ANOVA (followed by Tukey’s multiple comparison test) or unpaired *t* test was used for normally distributed data points and a Kruskal–Wallis (followed by Dunn’s multiple comparison test) or Mann–Whitney test was applied for measurements that did not follow normal distribution. The statistical tests used for each analysis are mentioned in the results section. For both parametric and nonparametric datasets, a significance level of 5% was chosen and accordingly the *p* values are displayed as follows: *p* < 0.05 (*); *p* < 0.01 (**), *p* < 0.001 (***).

## Results

### Variable cerebellar B cell and T cell pathology in MP4-immunized mice

**Fig. 2:**
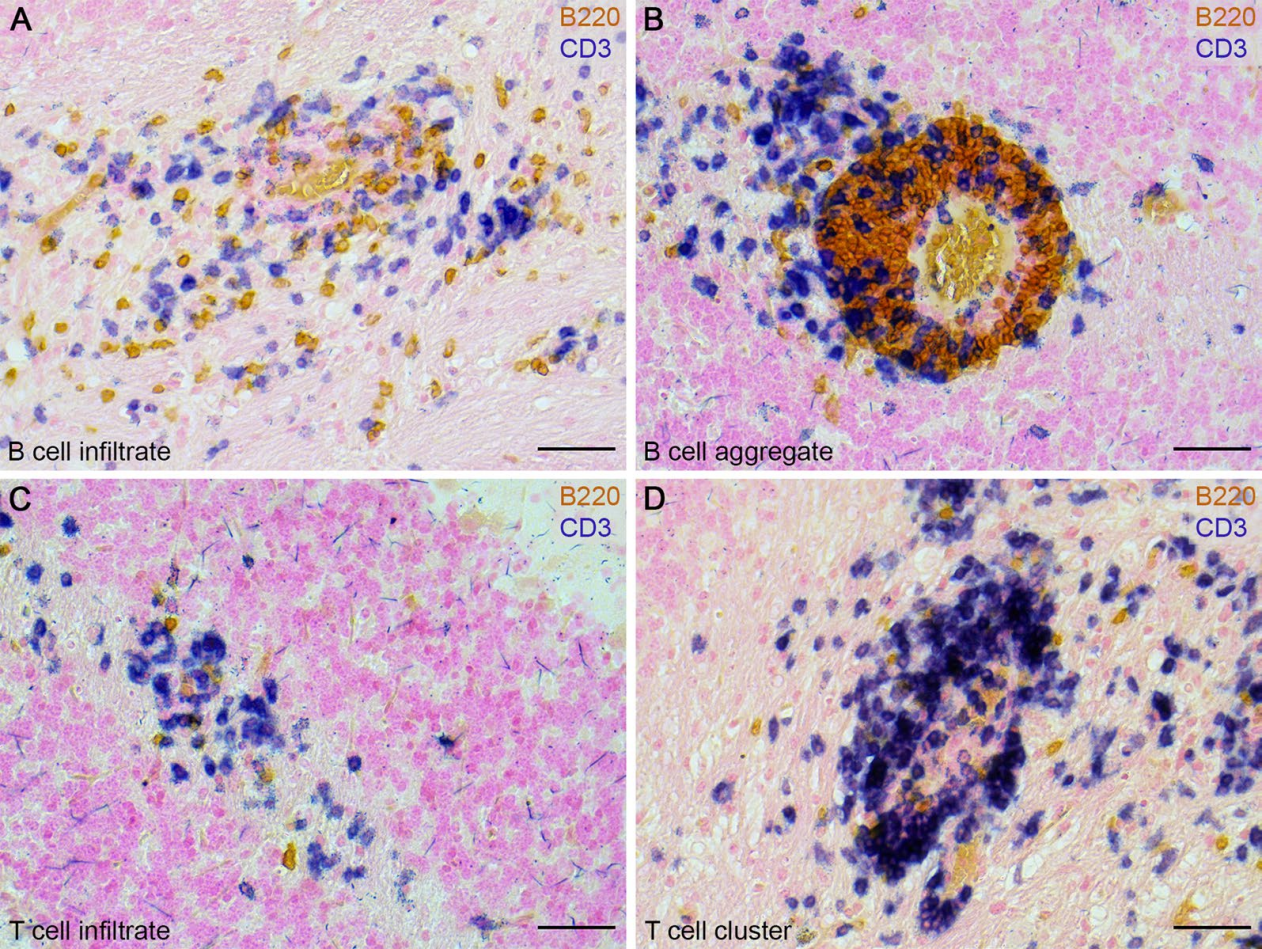
B cell and T cell pathology in cerebellar tissue of MP4-immunized mice.   The pathological classification is shown. Next to **A** B cell infiltrates and **B** B cell aggregates, mice developed **C** T cell infiltrates and **D** T cell clusters. The scale bars represent 50 µm

To evaluate the extent of B cell and T cell infiltration, cerebella of 31 MP4-immunized mice were screened for B220^+^ B cells and CD3^+^ T cells during the chronic stage of the disease. The mean of the disease onset was 26.52 ± 2.29 days and mice were killed 66.13 ± 1.83 days after immunization. The mean of the EAE score of the mice was 0.98 ± 0.16. Vehicle mice (control group immunized with CFA and PBS without antigen) were killed 62.00 ± 0.00 days after immunization and did not show any symptoms. MP4-immunized mice were categorized into different groups based on the number of cerebellar B cell infiltrates and aggregates or T cell infiltrates and clusters during the chronic disease stage. Representative images of the four main categories (i.e., B cell infiltrates, T cell infiltrates, B cell aggregates and T cell clusters) are shown in Fig. 2. A detailed summary of the different groups based on the B cell and T cell pathology is listed in Table 2. Groups of mice with a minimum of *n* = 3 were used for further analysis. Therefore, groups showing the following criteria were excluded from the study: no B cell and T cell pathology, no B cell and moderate T cell pathology, high B cell and moderate T cell pathology. Furthermore, an additional comparison was done between groups of mice with more than 30 B cell aggregates in the cerebellar tissue and those with no B cell aggregates as shown in Table 3.

**Table 2:** Experimental groups based on B cell and T cell pathology in MP4-immunized mice and controls

Groups	Criteria
Control (*n* = 5)	No B cell and T cell infiltration
Moderate B cell pathology/moderate T cell pathology (*n* = 11)	Number of B cell infiltrates + B cell aggregates: 1–49 Number of T cell infiltrates + T cell clusters: 1–49
Moderate B cell pathology/high T cell pathology (*n* = 6)	Number of B cell infiltrates + B cell aggregates: 1–49 Number of T cell infiltrates + T cell clusters: ≥ 50
High B cell pathology/high T cell pathology (*n* = 8)	Number of B cell infiltrates + B cell aggregates: ≥ 50 Number of T cell infiltrates + T cell clusters: ≥ 50

**Table 3:** Experimental groups based on B cell aggregation in MP4-immunized mice

Groups	Criteria
No B cell aggregates (*n* = 4)	Number of B cell aggregates: 0 Number of B cell and/or T cell infiltrates: ≥ 3
High amount of B cell aggregates (*n* = 6)	Number of B cell aggregates: > 30

### Differential expression of chemokines and cytokines in the CSF of MP4-immunized mice

**Fig. 3:**
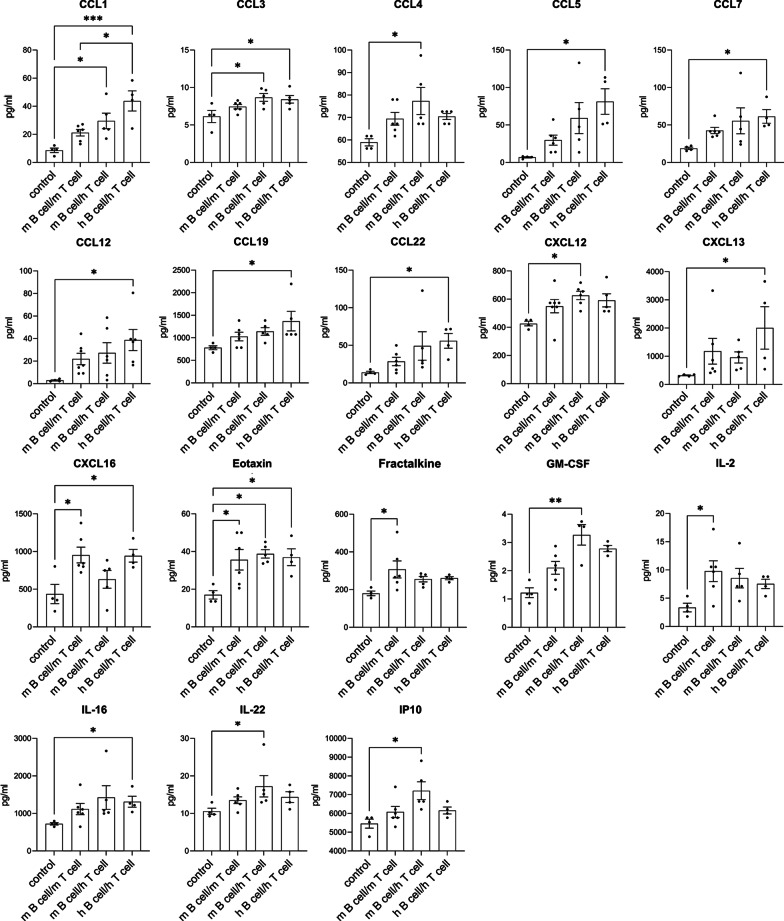
Cytokine and chemokine concentrations in the CSF comparing different groups of MP4-immunized mice. The comparison of the cytokine and chemokine concentration (pg/ml) in the “m B cell/m T cell”, the “m B cell/h T cell”, “h B cell/h T cell” groups and the controls is displayed. Only those chemokines and cytokines showing a significant difference in the concentration between the different groups are displayed. Mean values ± SEM are shown. *p < 0.05, **p < 0.01, ***p < 0.001. One-way ANOVA (normal distribution), Kruskal–Wallis test (no normal distribution). *CCL* C-C motif chemokine ligand, *CXCL* C-X-C motif chemokine ligand, *GM-CSF* granulocyte–macrophage colony-stimulating factor, *h B cell/h T cell* high B cell and T cell pathology, *IL* interleukin, *IP10* interferon-γ-induced protein 10, *m B cell/h T cell* moderate B cell and high T cell pathology, *m B cell/m T cell* moderate B cell and T cell pathology, *SEM* standard error of the mean

The levels of 34 cytokines and chemokines were quantified in the CSF of MP4-immunized mice that were categorized based on the extent of their cerebellar B cell and T cell pathology (Table 2). 18 of these cytokines and chemokines were elevated in the CSF of MP4-immunized mice compared to vehicle mice (Fig. 3).

The concentration of fractalkine and IL-2 was higher in CSF samples of the “moderate B cell and T cell pathology” (m B cell/m T cell) group compared to controls (*p* < 0.05), while CXCL16 was additionally significantly elevated in the “high B cell and T cell pathology” (h B cell/h T cell) group compared to controls (*p* < 0.05). Furthermore, CCL5, CCL7, CCL12, CCL19, CCL22, CXCL13 and IL-16 were significantly higher in the “h B cell/h T cell” group in comparison to vehicle mice (*p* < 0.05). In addition to CCL4, CXCL12, granulocyte–macrophage colony-stimulating factor (GM-CSF), IL-22 and interferon-γ-induced protein 10 kDa (IP10), which were significantly elevated in the “moderate B cell and high T cell pathology” (m B cell/h T cell) group (*p* < 0.05, *p* < 0.01), CCL3 showed a significantly higher concentration in the “m B cell/h T cell” and the “h B cell/h T cell” compared to the control group (*p* < 0.05). Moreover, eotaxin was significantly elevated in all three pathological groups in comparison to controls (*p* < 0.05). In addition to a significantly increased level of CCL1 in the “m B cell/h T cell” and the “h B cell/h T cell” group compared to vehicle mice (*p* < 0.05, *p* < 0.001), this chemokine was also significantly upregulated in the “h B cell/h T cell” group compared to the “m B cell/m T cell” group (*p* < 0.05).

### A specific chemokine signature is associated with B cell aggregation in MP4-immunized mice

**Fig. 4:**
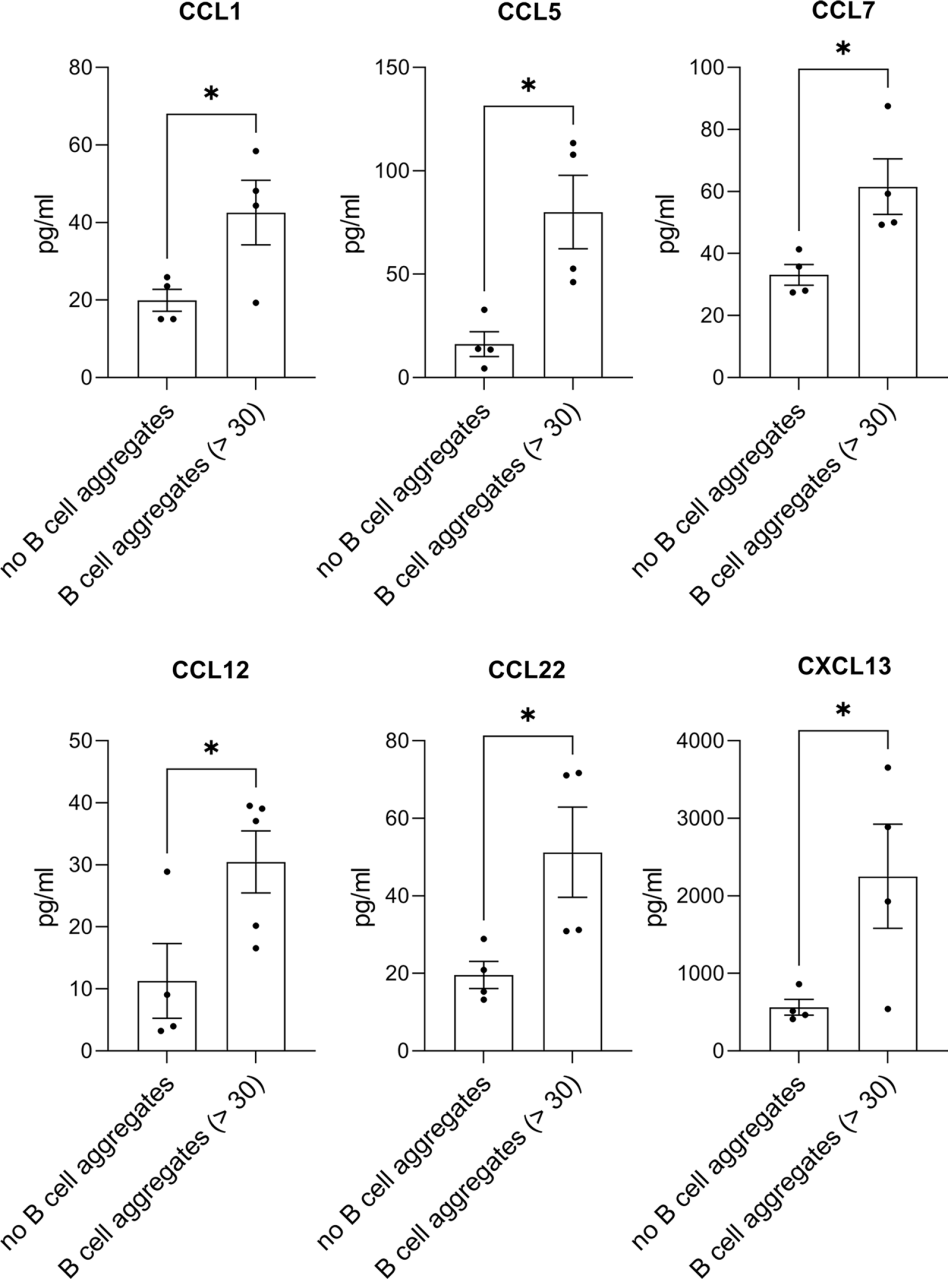
Cytokine and chemokine concentrations in the CSF comparing MP4-immunized mice with or without B cell aggregates. The graphs display the cytokine and chemokine concentrations (pg/ml) in the CSF of MP4-immunized mice showing B cell aggregates (> 30 B cells) compared to mice without aggregation. Chemokines and cytokines with significant differences between the two groups are presented. Mean values ± SEM are shown.  *p < 0.05, unpaired t-test (normal distribution), Mann–Whitney test (no normal distribution), *CCL* C-C motif chemokine ligand, *CXCL* C-X-C motif chemokine ligand, *SEM* standard error of the mean

CCL1, CCL5, CCL7, CCL12, CCL22 and CXCL13 were significantly higher in the group with more than 30 B cell aggregates compared to the group without any B cell aggregates (*p* < 0.05) (Fig. 4).

### CXCL16 expression in the serum is associated with the absence of B cell aggregates in MP4-immunized mice

Although there were no significant differences in the serum concentration of most of the cytokines and chemokines tested between the different groups, CXCL16 was significantly elevated in the serum of MP4-immunized mice that did not display any B cell aggregates in the cerebellum compared to those with a high amount of B cell aggregates (*p* < 0.05) (Additional file 1).

Comparative values between groups, which did not reach the significance level in CSF or serum, are shown in Additional file 1.

### CXCR5 and CXCR6 are preferentially expressed in the cerebellum of MP4-immunized mice with high B cell and T cell pathology

Cerebellar tissue of mice from the different groups was additionally screened for the expression pattern of three specific chemokine receptors whose corresponding chemokines were significantly upregulated in the CSF of MP4-immunized mice. These receptors were chosen based on their association with the formation and maintenance of ELS (Table 4) [14, 20, 21].

**Table 4:** Chemokine receptors and their reacting chemokines

Receptor	Chemokine
CCR5	e.g., CCL3, CCL4, CCL5
CXCR5	CXCL13
CXCR6	CXCL16

**Fig. 5:**
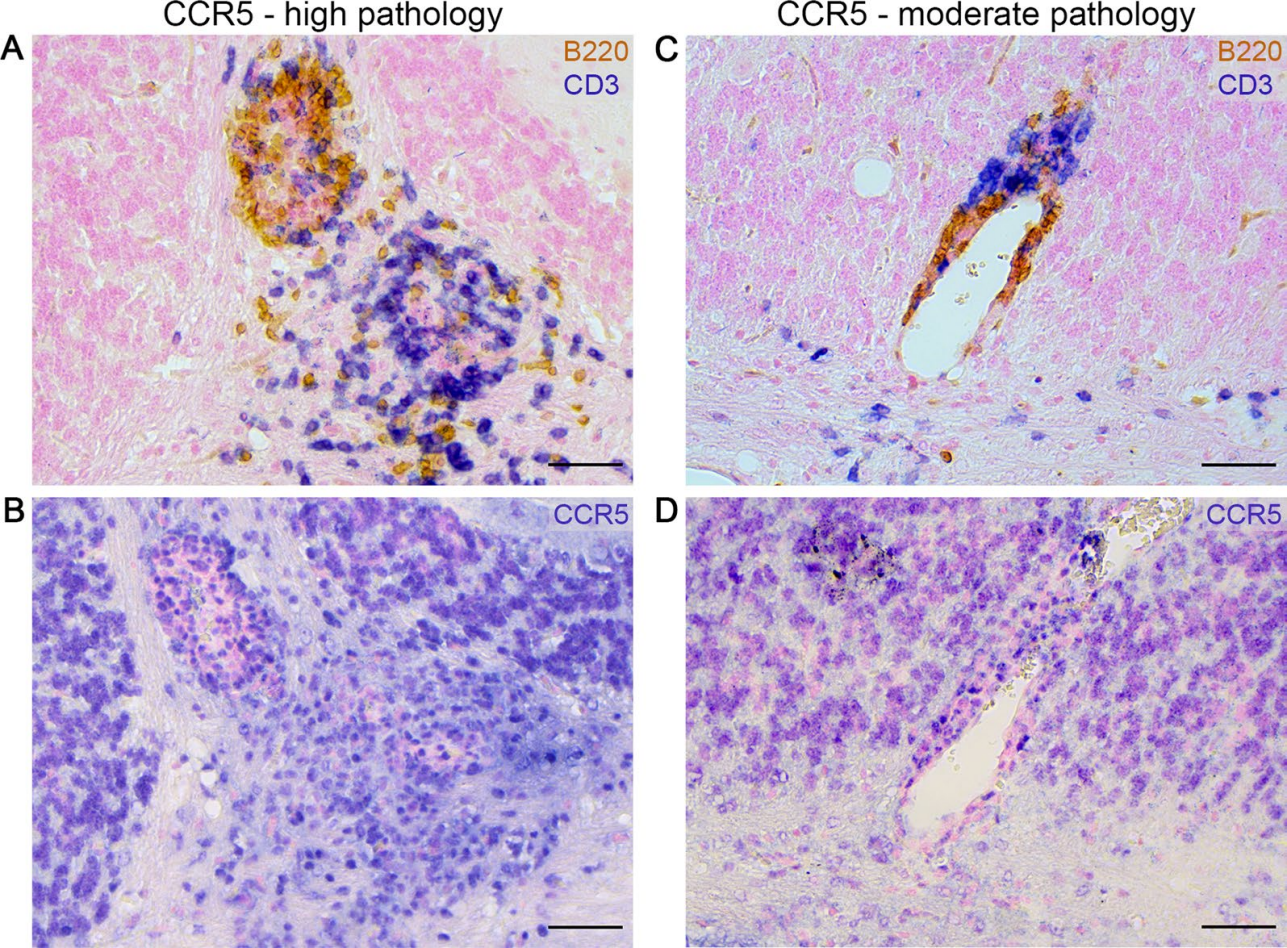
Expression of the chemokine receptor CCR5 in cerebellar tissue of MP4-immunized mice. In addition to **A** high B cell and T cell pathology and **B** its corresponding CCR5 expression, **C** moderate B cell and T cell pathology, and the associated **D** CCR5 expression is shown. Scale bars represent 50 µm. *CCR* C-C motif chemokine receptor

**Fig. 6:**
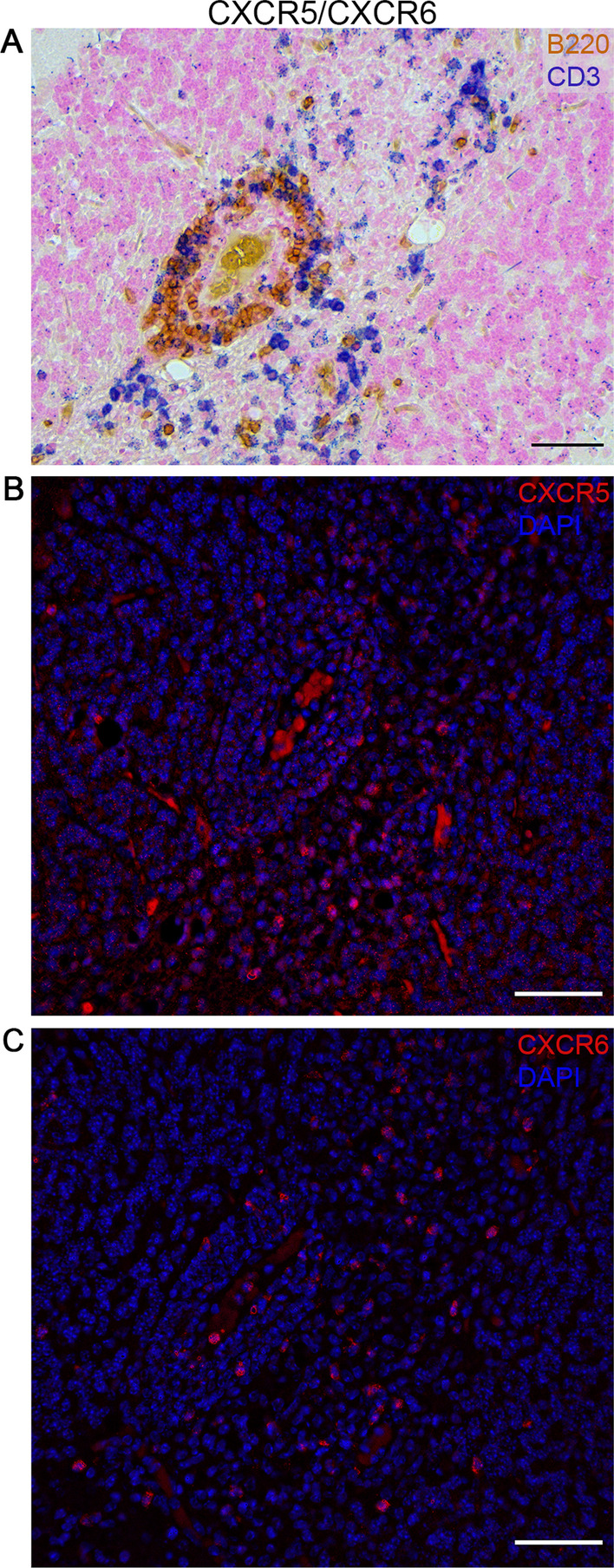
Expression of the chemokine receptors CXCR5 and CXCR6 in cerebellar tissue of MP4-immunized mice. **A** B cell and T cell pathology and the corresponding expression of **B** CXCR5 and **C** CXCR6 is displayed. Scale bars represent 50 µm. *CXCR* C-X-C motif chemokine receptor, *DAPI* 4',6-diamidino-2-phenylindole

**Fig. 7:**
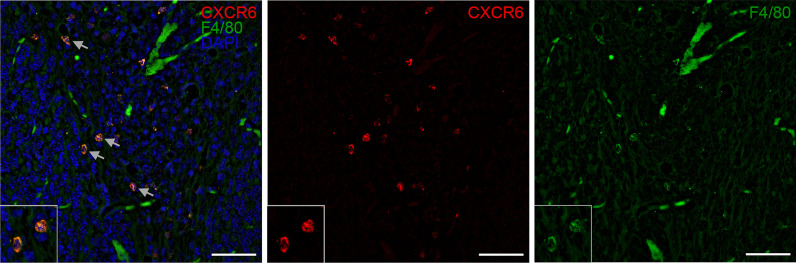
CXCR6-expressing macrophages. Representative images of CXCR6^+^F4/80^+^ macrophages are displayed. Double positive cells are marked with grey arrows and a magnified image of two relevant cells is shown. Scale bars represent 50 µm. *CXCR* C-X-C motif chemokine receptor, *DAPI* 4’,6-diamidino-2-phenylindole

While cerebellar expression of CCR5 was observed independent of the extent of B cell and T cell pathology in MP4-immunized mice, the presence of CXCR5 was mostly restricted to areas of high T cell infiltration and was mostly detected in the “h B cell/h T cell" group as well as in mice with a high number of B cell aggregates (Table 5, Fig. 5, Fig. 6). The same groups also showed an expression of CXCR6 which was primarily restricted to areas containing B cell aggregates in spatial association with infiltrates or only T cell-dominated infiltrates (Fig. 6). Additionally, we identified F4/80^+^ macrophages to express CXCR6 in the cerebellar tissue (Fig. 7), while T cells and B cells could be excluded as a source for CXCR5.

**Table 5:** Expression of chemokine receptors in the cerebellum of MP4-immunized mice

Groups	CCR5 [positive sections/total number of sections]	CXCR5 [positive sections/total number of sections]	CXCR6 [positive sections/total number of sections]
Moderate B cell/moderate T cell	5/6	1/6	2/7
Moderate B cell/high T cell	4/4	0/4	2/5
High B cell/high T cell	5/5	4/4	4/5
No B cell aggregates	2/3	0/3	1/3
High number of B cell aggregates (> 30)	4/4	3/4	4/5

## Discussion

Analysis of CSF and serum samples plays an important role in investigating the development and progression of pathophysiological processes in different diseases. In the case of neuroinflammatory disorders like MS, changes in chemokine and cytokine concentration, especially in the CSF, can be correlated to disease progression and CNS pathology [7, 22].

Given the restrictions of obtaining paired CSF and tissue samples from MS patients containing ELS, in this study we used the MP4-induced EAE model to identify a specific chemokine/cytokine signature associated with B cell and T cell pathology, with a particular focus on B cell aggregates. To this end, paired CSF and serum levels of an array of different cytokines and chemokines were compared between different groups of MP4-immunized mice that were stratified according to their cerebellar pattern of B cell and T cell pathology. Our findings indicate that a range of molecules were significantly upregulated especially in the CSF of MP4-immunized compared to control mice.

For example, CSF levels of CXCL16 were significantly higher in two groups, the “m B cell/m T cell” group and the “h B cell/h T cell” group. The importance of this chemokine in the formation of ELS and T cell accumulation has been previously described [20, 23]. CXCL16 is highly expressed in the CNS during pathological conditions such as in MS [24] and its specific receptor CXCR6 is primarily expressed on T cells [25–28], but also on microglial cells [29]. We did not detect CXCR6^+^ T cells in the cerebellum of the MP4-immunized mice. In our model, F4/80^+^ macrophages were responsible for CXCR6 expression, which is similar to findings in patients with glioblastoma [29].

Moreover, fractalkine levels are known to be increased in the CSF of patients with clinically isolated syndrome and are believed to be involved in the recruitment of CX3CR1^+^ CD4^+^ T cells into the CNS in early stages of MS [30]. However, we found an increase in the concentration of fractalkine in the CSF of mice with “m B cell/ m T cell pathology” compared to controls in the chronic stage of MP4-immunized EAE. Therefore, an influence of this chemokine at a later stage of the disease is conceivable. One of the interesting candidates that was significantly elevated in all three pathological groups in comparison to controls was eotaxin. Eotaxins are known to facilitate eosinophil recruitment to sites of inflammation [31]. In the context of MS, an elevation of eotaxin-1 (CCL11) in the CSF and plasma was associated with disease progression and severity especially in patients with secondary progressive disease [32]. Which target cell population is responsive to this increase in eotaxin in the CSF in the MP4-induced EAE model and what role this chemokine plays remain to be clarified.

Another interesting candidate was CCL1, which was also the only chemokine whose expression levels significantly correlated with the extent of B cell and T cell pathology. One can assume a protective function of CCL1 in the MP4-induced EAE model, due to its involvement in the proliferation of regulatory T cells which are known to secrete anti-inflammatory cytokines like IL-10 [33].

Additionally, the chemokines CCL1, CCL5, CCL7, CCL12, CCL22 and CXCL13 were associated with the presence of cerebellar B cell aggregates in MP4-immunized mice. The role of CXCL13 in the recruitment of B cells and follicular helper T cells has been extensively described for both MS and EAE [5, 7, 13, 14, 34–37]. Although these studies indicate a potential role of this chemokine in the formation of ELS by recruiting immune cells to the site of inflammation [10, 38], we did not detect any CXCR5 expression neither on B cells nor T cells in the cerebellum of MP4-immunized mice. In addition to CXCL13 it is conceivable that CCL5 may also be involved in the recruitment of immune cells into the CNS in MP4-immunized mice. EAE induced in CCR5-knockout mice showed a decrease in infiltrating immune cells indicating an important role of CCL5 in cell recruitment into the CNS [39]. Moreover, CCL5 is thought to be important for leukocyte extravasation, thus facilitating the migration of these cells into the CNS [40] and has also been described to be responsible for recruiting monocytes and memory T cells in rheumatoid arthritis (RA) [41, 42]. Interestingly, CCL5 is associated with a chemokine profile which predicts the presence of ELS in colorectal cancer [21]. However, given that its receptor CCR5 was expressed in the cerebellar tissue independent of the presence of B cell aggregates, its function in MP4-induced EAE might not be restricted to ELS formation and maintenance. CCL7 is another candidate that is involved in the formation of ELS in a mouse model of atherosclerosis [43]. In EAE, blockade of CCL7 led to a decreased migration of leukocytes [44]. Next to CCL12, which has been described to be involved in EAE development [45], CCL22 has also been shown to play a role in EAE by recruiting T cells and macrophages [46–48]. Additionally, in breast cancer CCL22 is known to influence the recruitment of regulatory T cells to areas of immune cell infiltrates [49]. Regarding its role in the development of ELS, the level of CCL22 was shown to be elevated in ELS^+^ patients with an autoimmune disease of the thyroid gland [50].

In addition to the analysis of CSF, we also examined paired serum samples. Despite an elevated (pro)inflammatory profile in the CSF, as discussed above, we noticed minimal alterations in the cytokine and chemokine expression pattern in the serum of MP4-immunized mice compared to vehicle. This observation can be explained by the fact that the CNS/CSF compartment might be conducive for immune cell recruitment, proliferation and differentiation independent of the periphery in the chronic stage of the disease [14, 17]. Interestingly, the level of CXCL16 was significantly increased in the serum of mice without cerebellar B cell aggregates compared to those with aggregates. It can be speculated that mice with B cell aggregates fostered a more local and compartmentalized environment for the survival and differentiation of immune cells than mice without these aggregates. Therefore, inflammation might have been more trapped in mice with B cell aggregates without the need for immune cell trafficking from the periphery. However, precisely why CXCL16 was the only chemokine that was upregulated in the serum cannot be explained by our current findings.

## Conclusion

There is a paucity of studies focusing on the molecular signature involved in ELS formation and maintenance in the CNS of mice due to the challenging nature of CSF collection. Our data provide a comprehensive overview of several chemokine and cytokine candidates that were upregulated in the CSF and may be involved in the development and maintenance of B cell aggregates. While future studies need to validate our findings using a higher number of mice, our current findings point towards a distinct profile of chemokines that can be used to predict cerebellar B cell aggregation in the chronic stage of MP4-induced EAE. Regarding the occurrence of ELS in MS patients, the identification of a specific chemokine signature in human CSF would be highly beneficial for *ante mortem* studies of these structures.

## Supplementary Information

**Additional file 1: Fig. S1.** Chemokine receptor staining and corresponding negative controls. Representative images of **A** a negative control using only secondary antibody only and its corresponding **B** CCR5 staining is displayed. Furthermore, the **C** negative control for **D** CXCR5 and **E** CXCR6 staining is shown. Scale bars represent 50 µm. *CCR* C-C motif chemokine receptor, *CXCR* C-X-C motif chemokine receptor, *DAPI *4',6-diamidino-2-phenylindole. **Fig. S2.** CXCL16 concentration in the serum comparing MP4-immunized mice with or without B cell aggregates. The significant increase in the CXCL16 concentration (pg/ml) in the serum of MP4-immunized mice without B cell aggregates compared to mice showing B cell aggregates (> 30 B cells) is displayed. Mean values ± SEM are shown.  *p < 0.05, unpaired *t*-test, *CXCL* C-X-C motif chemokine ligand, *SEM* standard error of the mean. **Table S1.** Non-significant data of cytokines and chemokines in the CSF of different pathology groups. **Table S2.** Non-significant data of cytokines and chemokines in the CSF with reference to B cell aggregates. **Table S3.** Non-significant data of cytokines and chemokines in the serum of different pathology groups. **Table S4.** Non-significant data of cytokines and chemokines in the serum with reference to B cell aggregates.

## Data Availability

The datasets used and/or analyzed during the current study are available from the corresponding author on reasonable request.

## Abbreviations

AP: Alkaline phosphatase
ARRIVAL: Animal Research: Reporting of In Vivo Experiments
B6: C57BL/6
BAFF: B-cell activating factor of the TNF family
BSA: Bovine serum albumin
CCL: C-C motif chemokine ligand
CCR: C-C motif chemokine receptor
CFA: Complete Freund´s adjuvant
CNS: Central nervous system
CO_2_: Carbon dioxide
CSF: Cerebrospinal fluid
CV: Coefficient of variation
CXCL: C-X-C motif chemokine ligand
CXCR: C-X-C motif chemokine receptor
DAPI: 4',6-Diamidino-2-phenylindole
EAE: Experimental autoimmune encephalomyelitis
ELS: Ectopic lymphoid structures
FAU: Friedrich-Alexander-Universität Erlangen-Nürnberg
FDC: Follicular dendritic cells
FPZ: Franz-Penzoldt Center
GM-CSF: Granulocyte–macrophage colony-stimulating factor
h B cell/h T cell: High B cell and T cell pathology
HRP: Horseradish peroxidase
IL: Interleukin
IFN: Interferon
IFA: Incomplete Freund’s adjuvant
IP10: Interferon-γ-induced protein 10 kDa
LT: Lymphotoxin
m B cell/h T cell: Moderate B cell and high T cell pathology
m B cell/m T cell: Moderate B cell and T cell pathology
MS: Multiple sclerosis
PBS: Phosphate-buffered saline
PETZ: Preclinical Experimental Center for Animals
PFA: Paraformaldehyde
PO_4_: Phosphate
RA: Rheumatoid arthritis
RRMS: Relapsing–remitting multiple sclerosis
RT: Room temperature
SEM: Standard error of the mean
SLO: Secondary lymphoid organs
SPMS: Secondary progressive multiple sclerosis
TBS: Tris-buffered saline
TBS-T: TBS + 0.1% Tween 20
TNF: Tumor necrosis factor
TRC: Translational Research Center
WT: Wild-type
