# Supplementary material for: The presence of cerebellar B cell aggregates is associated with a specific chemokine profile in the cerebrospinal fluid in a mouse model of multiple sclerosis
Source: J Neuroinflammation. 2023 Jan 30;20:18. doi: 10.1186/s12974-023-02695-z (PMC9885581; doi:10.1186/s12974-023-02695-z)
Supplement: Supplementary file 1 — Additional file 1: Fig. S1. Chemokine receptor staining and corresponding negative controls. Representative images of A a negative control using only secondary antibody only and its corresponding B CCR5 staining is displayed. Furthermore, the C negative control for D CXCR5 and E CXCR6 staining is shown. Scale bars represent 50 µm. CCR C-C motif chemokine receptor, CXCR C-X-C motif chemokine receptor, DAPI 4',6-diamidino-2-phenylindole. Fig. S2. CXCL16 concentration in the serum comparing MP4-immunized mice with or without B cell aggregates. The significant increase in the CXCL16 concentration (pg/ml) in the serum of MP4-immunized mice without B cell aggregates compared to mice showing B cell aggregates (> 30 B cells) is displayed. Mean values ± SEM are shown. *p < 0.05, unpaired t-test, CXCL C-X-C motif chemokine ligand, SEM standard error of the mean. Table S1. Non-significant data of cytokines and chemokines in the CSF of different pathology groups. Table S2. Non-significant data of cytokines and chemokines in the CSF with reference to B cell aggregates. Table S3. Non-significant data of cytokines and chemokines in the serum of different pathology groups. Table S4. Non-significant data of cytokines and chemokines in the serum with reference to B cell aggregates. [file 12974_2023_2695_MOESM1_ESM.docx]

**Additional file 1**


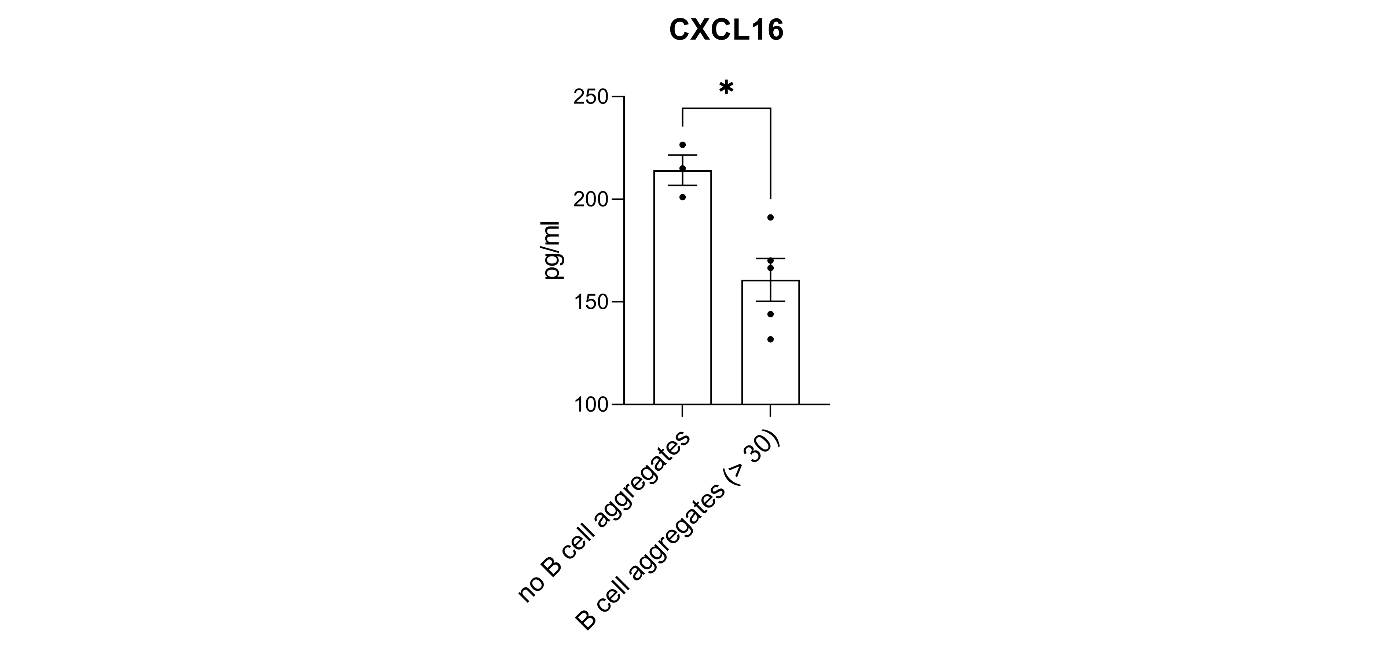


**Fig. S1** CXCL16 concentration in the serum comparing MP4-immunized mice with or without B cell aggregates. The significant increase in the CXCL16 concentration (pg/ml) in the serum of MP4-immunized mice without B cell aggregates compared to mice showing B cell aggregates (> 30 B cells) is displayed. **p* < 0.05, unpaired t-test, CXCL = C-X-C motif chemokine ligand.


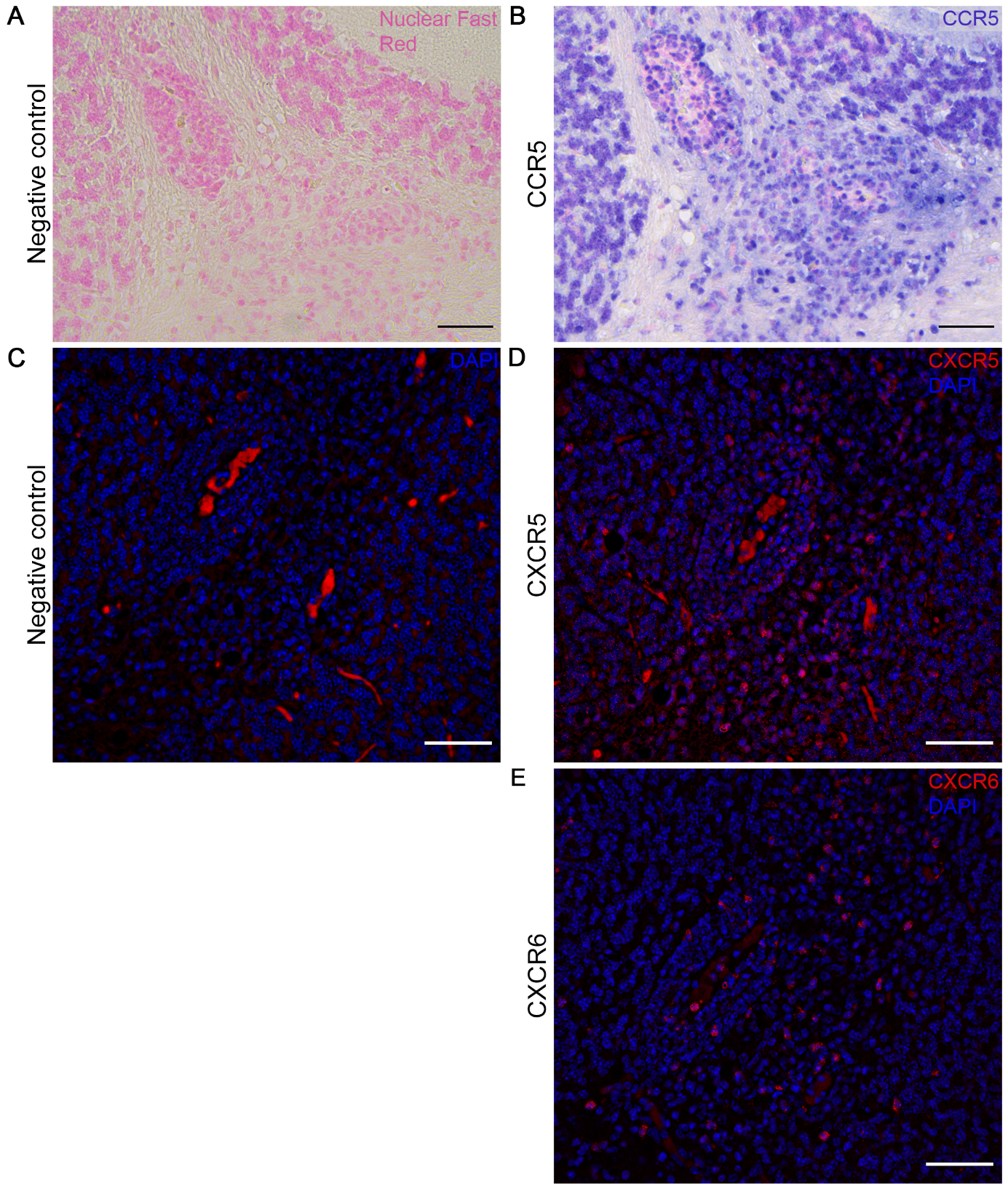


**Fig. S2** Chemokine receptor staining and corresponding negative controls. Representative images of **A** a negative control using only secondary antibody and its corresponding **B** CCR5 staining is displayed. Furthermore, the **C** negative control for **D** CXCR5 and **E** CXCR6 staining is shown. Scale bars represent 50 µm. CCR = C-C motif chemokine receptor, CXCR = C-X-C motif chemokine receptor, DAPI = 4',6-diamidino-2-phenylindole.

**Table S1** Non-significant data of cytokines and chemokines in CSF of different pathology groups

| **Cytokine/chemokine** | **Control**  **[pg/ml]** | **m B cell/m T cell**  **[pg/ml]** | **m B cell/h T cell**  **[pg/ml]** | **h B cell/h T cell**  **[pg/ml]** |
| --- | --- | --- | --- | --- |
| CCL2 | 182.74 ± 26.80 | 220.16 ± 39.37 | 272.64 ± 20.96 | 284.55 ± 18.93 |
| CCL17 | 51.57 ± 1.51 | 68.12 ± 5.78 | 81.17 ± 13.07 | 76.92 ± 4.70 |
| CCL20 | 46.31 ± 2.68 | 58.72 ±7.81 | 61.94 ± 4.98 | 58.35 ± 1.69 |
| CCL27 | 1604.83 ± 383.57 | 3041.16 ± 896.66 | 3189.13 ± 656.19 | 2332.56 ± 151.25 |
| CXCL1 | 45.33 ± 2.90 | 61.98 ± 7.46 | 75.81 ± 13.67 | 88.34 ± 13.32 |
| CXCL5 | 351.00 ± 26.14 | 353.37 ± 17.07 | 451.92 ± 38.59 | 394.76 ± 28.45 |
| CXCL11 | 1589.61 ± 245.08 | 2288.45 ± 100.55 | 2380.82 ± 393.78 | 2176.03 ± 112.42 |
| Eotaxin-2 | 66.05 ± 66.05 | 721.96 ± 186.94 | 601.72 ± 196.27 | 940.84 ± 370.03 |
| IFN-γ | 40.06 ± 3.97 | 46.78 ± 4.59 | 53.66 ± 4.55 | 46.59 ± 3.77 |
| IL-1β | 231.51 ± 68.07 | 328.52 ± 18.69 | 383.50 ± 44.42 | 346.53 ± 18.65 |
| IL-4 | 53.63 ± 6.00 | 60.35 ± 2.27 | 71.51 ± 5.32 | 62.22 ± 4.48 |
| IL-6 | 11.34 ± 1.57 | 39.06 ± 21.34 | 39.68 ± 19.77 | 26.61 ± 1.92 |
| IL-10 | 282.31 ± 44.30 | 418.74 ± 38.42 | 460.72 ± 57.43 | 448.79 ± 23.47 |
| IL-21 | 995.72 ± 520.66 | 1795.91 ± 58.05 | 1793.70 ± 297.02 | 1423.95 ± 169.94 |
| IL-23 | 4962.01 ± 359.66 | 4176.89 ± 472.71 | 5254.49 ± 1040.33 | 4002.35 ± 94.63 |
| TNF-α | 334.57 ± 9.13 | 354.62 ± 23.44 | 389.28 ± 50.34 | 319.40 ± 22.16 |

Mean values ± SEM are shown. CCL = C-C motif chemokine ligand, CXCL = C-X-C motif chemokine ligand, IFN = interferon, IL = interleukin, m B cell/m T cell = moderate B cell pathology and moderate T cell pathology, m B cell/ h T cell = moderate B cell and high T cell pathology, h B cell/ h T cell = high B cell and high T cell pathology, TNF = tumor necrosis factor.

**Table S2** Non-significant data of cytokines and chemokines in CSF regarding B cell aggregates

| **Cytokine/chemokine** | **No aggregates**  **[pg/ml]** | **High amount of aggregates**  **[pg/ml]** |
| --- | --- | --- |
| CCL2 | 280.84 ± 23.79 | 264.74 ± 19.03 |
| CCL3 | 7.61 ± 0.78 | 7.69 ± 0.46 |
| CCL4 | 75.47 ± 7.57 | 67.18 ± 2.22 |
| CCL17 | 61.78 ± 4.60 | 75.23 ± 5.07 |
| CCL19 | 1008.92 ± 85.09 | 1219.38 ± 86.99 |
| CCL20 | 66.62 ± 12.52 | 55.25 ± 4.45 |
| CCL27 | 3768.09 ± 1169.55 | 2180.82 ± 143.08 |
| CXCL1 | 63.88 ± 6.03 | 74.16 ± 7.14 |
| CXCL5 | 404.14 ± 52.97 | 360.34 ± 29.39 |
| CXCL11 | 2305.91 ± 140.62 | 1935.87 ± 127.74 |
| CXCL12 | 611.23 ± 68.12 | 542.61 ± 31.12 |
| CXCL16 | 719.44 ± 243.37 | 1016.3 ± 70.94 |
| Eotaxin | 33.80 ± 5.85 | 39.82 ± 4.84 |
| Eotaxin-2 | 672.64 ± 199.62 | 770.18 ± 255.73 |
| Fractalkine | 301.42 ± 69.71 | 286.68 ± 19.19 |
| GM-CSF | 2.33 ± 0.47 | 2.49 ± 0.34 |
| IFN-γ | 56.74 ± 5.59 | 44.41 ± 3.08 |
| IL-1β | 329.74 ± 35.00 | 320.30 ± 11.56 |
| IL-2 | 9.20 ± 2.91 | 6.67 ± 0.83 |
| IL-4 | 66.77 ± 7.54 | 56.66 ± 4.19 |
| IL-6 | 16.78 ± 2.42 | 24.31 ± 3.46 |
| IL-10 | 453.12 ± 57.67 | 444.66 ± 29.83 |
| IL-16 | 1108.01 ± 235.45 | 1248.48 ± 171.65 |
| IL-21 | 2096.65 ± 275.21 | 1337.54 ± 172.09 |
| IL-22 | 13.67 ± 1.15 | 13.67 ± 1.78 |
| IL-23 | 4917.84 ± 1269.20 | 3791.59 ± 465.77 |
| IP10 | 6998.79 ± 705.01 | 5791.51 ± 390.86 |
| TNF-α | 384.77 ± 62.20 | 294.99 ± 18.87 |

Mean values ± SEM are shown. CCL = C-C motif chemokine ligand, CXCL = C-X-C motif chemokine ligand, GM-CSF = granulocyte macrophage colony-stimulating factor, IFN = interferon, IL = interleukin, IP10 = interferon-γ induced protein 10 kD, TNF = tumor necrosis factor.

**Table S3** Non-significant data of cytokines and chemokines in serum of different pathology groups

| **Cytokine/chemokine** | **Control**  **[pg/ml]** | **m B cell/m T cell**  **[pg/ml]** | **m B cell/h T cell**  **[pg/ml]** | **h B cell/h T cell**  **[pg/ml]** |
| --- | --- | --- | --- | --- |
| CCL2 | 590.28 ± 40.27 | 559.06 ± 59.31 | 559.23 ± 35.89 | 724.79 ± 43.84 |
| CCL3 | 38.20 ± 2.14 | 37.93 ± 2.48 | 39.41 ± 2.23 | 37.94 ± 3.16 |
| CCL4 | 151.92 ± 7.61 | 158.85 ± 7.94 | 161.43 ± 9.97 | 153.26 ± 9.60 |
| CCL5 | 266.40 ± 141.38 | 128.10 ± 9.85 | 127.88 ± 1.48 | 132.12 ± 15.50 |
| CCL7 | 132.66 ± 19.66 | 100.66 ± 10.41 | 123.38 ± 4.78 | 113.23 ± 14.81 |
| CCL12 | 29.14 ± 4.70 | 30.64 ± 6.22 | 25.19 ± 4.00 | 31.68 ± 8.42 |
| CCL17 | 362.78 ± 32.62 | 422.13 ± 22.55 | 398.79 ± 35.19 | 424.17 ± 30.13 |
| CCL19 | 2253.89 ± 15.02 | 2334.79 ± 81.98 | 2413.66 ± 192.55 | 2437.69 ± 123.37 |
| CCL22 | 332.19 ± 43.82 | 355.70 ± 26.29 | 389.24 ± 20.77 | 359.60 ± 13.07 |
| CCL27 | 7005.56 ± 533.87 | 7146.62 ± 750.96 | 6215.51 ± 413.18 | 7692.77 ± 898.26 |
| CXCL1 | 359.03 ± 10.31 | 360.68 ± 21.64 | 357.97 ±32.06 | 390.74 ± 13.74 |
| CXCL11 | 8687.27 ± 673.62 | 8113.45 ± 923.71 | 8861.28 ± 47.25 | 9758.57 ± 580.41 |
| CXCL12 | 2739.24 ± 328.84 | 3080.01 ±180.35 | 3212.57 ± 286.66 | 3387.79 ± 319.85 |
| CXCL13 | 5797.69 ± 1485.47 | 3368.06 ± 521.15 | 4978.36 ± 1054.97 | 4219.11 ± 755.80 |
| CXCL16 | 220.85 ± 57.83 | 181.21 ± 21.02 | 213.51 ± 9.81 | 174.40 ± 10.16 |
| Eotaxin | 830.56 ± 82.28 | 968.58 ± 96.02 | 1075.86 ± 121.44 | 1170.80 ± 109.40 |
| Eotaxin-2 | 9345.95 ± 499.05 | 8577.34 ± 473.21 | 9073.94 ± 1021.38 | 7986.22 ± 649.36 |
| GM-CSF | 9.232 ± 0.47 | 9.50 ± 0.89 | 9.35 ± 0.30 | 10.24 ± 1.35 |
| IFN-γ | 233.81 ± 7.60 | 260.42 ±14.61 | 238.54 ± 15.64 | 302.29 ± 28.08 |
| IL-1β | 679.81 ± 46.45 | 736.73 ± 70.22 | 649.08 ± 50.77 | 786.38 ± 82.24 |
| IL-2 | 37.83 ± 7.49 | 60.29 ± 8.89 | 45.05 ± 7.31 | 66.23 ± 21.16 |
| IL-4 | 109.31 ± 13.75 | 121.82 ±11.05 | 116.57 ± 11.35 | 135.06 ± 11.93 |
| IL-6 | 78.60 ± 3.64 | 77.98 ±7.85 | 76.74 ± 5.64 | 84.75 ± 12.78 |
| IL-10 | 1711.08 ± 134.76 | 1663.20 ± 115.16 | 1886.34 ± 89.31 | 1694.05 ± 136.93 |
| IL-16 | 1150.89 ± 92.49 | 1177.40 ± 62.64 | 1176.89 ± 105.82 | 1383.29 ± 82.25 |
| IL-22 | 80.81 ± 7.58 | 70.62 ± 6.81 | 67.66 ± 3.97 | 75.67 ± 8.02 |
| IL-23 | 312.60 ± 224.87 | 521.60 ±450.37 | 10.92 ± 10.920 | 703.60 ± 367.90 |
| IP10 | 6204.17 ± 511.93 | 6121.22 ± 535.61 | 5345.43 ± 460.40 | 6867.45 ± 702.43 |
| TNF-α | 403.69 ± 55.36 | 486.36 ± 32.26 | 415.10 ± 22.73 | 489.41 ± 40.29 |

Mean values ± SEM is shown. CCL = C-C motif chemokine ligand, CXCL = C-X-C motif chemokine ligand, GM-CSF = granulocyte macrophage colony-stimulating factor, IFN = interferon, IL = interleukin, IP10 = interferon-γ induced protein 10 kD, m B cell/m T cell = moderate B cell pathology and moderate T cell pathology, m B cell/h T cell = moderate B cell and high T cell pathology, h B cell/h T cell = high B cell and high T cell pathology, TNF = tumor necrosis factor.

**Table S4** Non-significant data of cytokines and chemokines in the serum with reference to B cell aggregates

| **Cytokine/chemokine** | **No aggregates**  **[pg/ml]** | **High amount of aggregates**  **[pg/ml]** |
| --- | --- | --- |
| CCL1 | 58.68 ± 5.99 | 56.24 ± 18.76 |
| CCL3 | 36.07 ± 1.90 | 39.75 ± 4.14 |
| CCL4 | 153.64 ± 7.13 | 159.82 ± 12.16 |
| CCL17 | 420.60 ± 46.30 | 426.17 ± 32.43 |
| CCL19 | 2273.77 ± 178.99 | 2441.75 ± 107.08 |
| CCL20 | 51.23 ± 3.77 | 60.95 ±12.55 |
| CCL27 | 6877.33 ± 574.08 | 7974.50 ± 1468.41 |
| CXCL12 | 3791.14 ± 304.90 | 2966.23 ± 141.08 |
| CXCL13 | 4595.68 ± 896.22 | 4426.83 ± 618.24 |
| Eotaxin 2 | 9130.91 ± 471.66 | 8082.08 ± 856.13 |
| GM-CSF | 9.30 ±0.59 | 10.86 ± 1.74 |
| IL-1β | 677.42 ± 58.76 | 853.76 ± 119.36 |
| IL-2 | 45.59 ± 5.75 | 69.33 ± 20.58 |
| IL-4 | 120.76 ± 8.11 | 139.99 ± 16.22 |
| IL-6 | 76.62 ± 7.17 | 92.97 ±11.45 |
| IL-10 | 1749.82 ± 54.61 | 1661.78 ± 152.73 |
| IL-21 | 6464.83 ± 417.97 | 7058.52 ± 1309.65 |
| IL-22 | 72.99 ± 5.34 | 83.95 ± 9.49 |
| IL-23 | 14.56 ± 14.56 | 1236.82 ± 618.71 |
| IP10 | 6023.35 ± 300.37 | 7355.16 ± 962.22 |
| TNF-α | 425.92 ± 28.43 | 513.12 ± 35.13 |

Mean values ± SEM are shown. CCL = C-C motif chemokine ligand, CXCL = C-X-C motif chemokine ligand, GM-CSF = granulocyte macrophage colony-stimulating factor, IFN = interferon, IL = interleukin, IP10 = interferon-γ induced protein 10 kD, TNF = tumor necrosis factor.
